# Improving Training Condition Assessment in Endurance Cyclists: Effects of *Ganoderma lucidum* and *Ophiocordyceps sinensis* Dietary Supplementation

**DOI:** 10.1155/2014/979613

**Published:** 2014-04-01

**Authors:** Paola Rossi, Daniela Buonocore, Elisa Altobelli, Federico Brandalise, Valentina Cesaroni, Davide Iozzi, Elena Savino, Fulvio Marzatico

**Affiliations:** ^1^Department of Biology and Biotechnology, “L. Spallanzani,” Pavia University, Via Ferrata 9, 27100 Pavia, Italy; ^2^Department of Earth and Environmental Science, Pavia University, Pavia, Italy

## Abstract

The main reasons for taking daily dietary supplements are to maintain good health, to improve homeostasis, and to create conditions for reducing the risk of disease. Due to growing market demand, the search for effective, nontoxic, natural compounds with antioxidant and ergogenic properties has increasingly become a matter of interest. This paper describes how a specific combination of fungal supplements can help improve the performance of endurance athletes. We report the effects of a brief 3-month trial of two fungal supplements, *Ganoderma lucidum* and *Cordyceps sinensis* (3 capsules of *O. sinensis* and 2 capsules of *G. lucidum* per day), in 7 healthy male volunteers, aged between 30 and 40 years, who are all amateur cyclists that participate in “Gran Fondo” cycling races. This trial investigated the effects of fungal supplements on the level of physical fitness of the athletes by monitoring and comparing the following biomarkers just before and after physical exertion: the testosterone/cortisol ratio in the saliva and oxidative stress (DPPH free radical scavenging activity). A decrease of more than 30% in the testosterone/cortisol ratio after race compared to before race was considered as a risk factor for nonfunctional overreaching (NFO) or the overtraining syndrome (OTS). The results show that, after 3 months of supplementation, the testosterone/cortisol ratio changed in a statistically significant manner, thereby protecting the athletes from NFO and OTS. Antioxidant activity was measured by quantifying the scavenging ability of the human serum on the synthetic free radical DPPH. After 3 months of fungal supplementation, the data demonstrate an increased scavenger capacity of free radicals in the athletes' serum after the race, thereby protecting the athletes from oxidative stress.

## 1. Introduction

Fatigue and underperformance are common in athletes and can affect more than 65% at the peak of their career. The European College of Sport Science has defined these symptoms as nonfunctional overreaching (NFO) and the overtraining syndrome (OTS) [1–3]. Athletes increase their training load in order to improve their performance. However, a maladapted response to excessive exercise, without adequate rest, can lead to nonfunctional performance, such as a discrepancy between the stress incurred during training or competing and the athlete's ability to recover after exercise. The most commonly encountered effects are underperformance, reduced tolerance to training load, decreased coordination, and increased heart rate.

NFO is a performance decrement that requires 72 hours of recovery, while OTS is a performance decrement that requires days or weeks of recovery after maximal physical exercise [1–3]. It is clinically difficult to differentiate between NFO and OTS as the difference is based on the time it takes for athletes to recover and not necessarily on the degree or type of symptoms. Thus, a period of complete rest is often required before it is possible to diagnose one syndrome or the other. Many authors consider overreaching and overtraining as a continuum, while others suggest that NFO precedes OTS [3].

It would be very useful to understand the mechanisms underlying underperformance in order to assess, manage, and educate the athlete. The overtraining syndrome (OTS) disturbs many body systems (neurologic, endocrinologic, and immunologic systems). Many hypotheses have been presented to explain the pathogenesis of the OTS [2]. The hypothalamic hypothesis suggests that deregulation of the hypothalamus and hormonal axes activates the hypothalamic-pituitary-adrenal axis in endurance athletes, but data in the literature are contradictory. Furthermore, this hypothesis suggests that endurance athletes may experience changes in levels of cortisol, adrenocorticotropic hormone, testosterone, and other hormones. Unfortunately, literature data prove contradictory in terms of the pattern of hormonal changes involved [4–7]. One of the indices that is used to evaluate the stress induced by physical exercise is the ratio between the levels of testosterone and cortisol (T/C), both in basal conditions and after exercise. The T/C ratio is used to define the general metabolic trend in an organism: a decrease indicates a catabolic tendency, while an increase indicates an anabolic trend. In particular, data from the scientific literature show that a decrease of less than 30% in the T/C ratio indicates an effective workout, while a decrease of more than 30% from baseline in the T/C ratio is a risk factor for overtraining [7].

The oxidative stress hypothesis suggests that excessive oxidative stress causes muscle damage and fatigue. A small amount of data indicates that markers of oxidative stress are higher at rest in overtrained athletes and increase with exercise.

Many fungal species have recently been reported to display antioxidant and ergogenic properties, demonstrating what has already been claimed by Traditional Chinese Medicine (TCM) [8, 9]. One of the most popular fungi in this group is* Ganoderma lucidum* (Curtis) P. Karst., better known in western countries by the Japanese term “Reishi”; it is defined in Traditional Chinese Medicine (TCM) as the “mushroom of immortality.”* Ganoderma lucidum* preferentially grows on broadleaved trees, such as oak or chestnut, mainly as a saprotroph and only rarely as a parasite. This species has been reported to have multiple beneficial values because it contains a vast number of bioactive compounds, the most pharmacologically active of which being triterpenes and polysaccharides [10–14]. Another two of these bioactive compounds are adenosine monophosphate, thought to help lower blood pressure and remove blood vessel blockage, and superoxide dismutase, an antiaging substance. The main medicinal properties of this fungus are immune response modulating, anticancer, antihypertensive, antioxidant, antiaging, anti-inflammatory, antiviral, and cardio- and nephroprotection [15, 16].

Another important medicinal fungal species is* Ophiocordyceps sinensis* (Berk.) G. H. Sung, J. M. Sung, Hywel-Jones & Spatafora, more commonly known as* Cordyceps sinensis* (Berk.) Sacc. According to the last taxonomic revision, it is an ascomycete, endemic to the Tibetan Plateau and surrounding Himalayas. The fungus is parasitic and colonizes the larvae of a moth,* Hepialus armoricanus*, forming a complex that includes the remains of the parasitic larva and the stroma of the fungus (sclerotia). In its growth phase, the fungus gradually turns into a fruiting body.* Ophiocordyceps sinensis* is used in Traditional Chinese Medicine (TCM) to promote health, longevity, and athletic power because of its tonic effect, reducing fatigue. Recent studies have demonstrated that the chemical constituents extracted from this species have various protective effects on the nervous system, cardiovascular diseases, proliferation of tumor masses, lipid metabolism, and infection [17]; moreover, it has antiaging properties [18]. One of the constituents isolated from* O. sinensis,* cordycepin (3′-deoxyadenosine), is a derivative of the nucleoside adenosine, differing only in the absence of an oxygen molecule in the 3′ position of the ribose.

Athletes who perform endurance training undergo oxidative stress and potentially suffer from the overtraining syndrome. The aim of the present study was to evaluate the effects of two fungal supplements, used in TCM, on the athletic performance of road cyclists. Considering the different properties of the two fungal species mentioned above, we suggested that the athletes in our study group take this specific combination of dietary supplements.

## 2. Materials and Methods

### 2.1. Subjects

We selected a sample of 7 healthy male volunteers, aged between 30 and 40 years, who were all amateur cyclists and duly informed them of the procedures to be followed in the study. The subject age, weight, height, and BMI are reported in Table 1. All the subjects had been cycling for more than 10 years and usually performed about 300 km per week and 12,000/15,000 km per year. Each procedure was drawn up in agreement with the Helsinki Declaration adopted at the Eighteenth General Assembly of the World Medical Association (WMA), held in 1964 on ethical principles for medical research involving human subjects, and with the permission of the Ethic Committee of the University of Pavia, Italy.

The subjects were instructed not to change their lifestyle during the trial, including exercise, diet, and other routine activities, and not to take any other medicinal herbs or drugs. They were also instructed to report any adverse events to the investigators during the trial: none occurred.

### 2.2. Study Design

To assess the training condition of the athletes, according to the hypothalamic hypothesis, we evaluated the T/C ratio before and after race [7]: an increase in the after race value of more than 30% compared to the before race value was considered a risk factor for overtraining. Using saliva to calculate the levels of cortisol and testosterone is a valid alternative to using plasma: the concentration of cortisol and testosterone in saliva represents the hormone-free part and thus the biologically active component [19]. In order to test the oxidative stress hypothesis, we measured the free radical scavenging activity by DPPH.

We performed a double-blind clinical study. The volunteers took placebo supplements for the first month and then active supplements of* O*.* sinensis* and* G*.* lucidum* for the following 3 months. During the trial, the athletes performed daily workouts and took part in 2 “Gran Fondo” cycling races. During the placebo phase, we monitored the athletes before and after the first race, Gran Fondo Laigueglia, with a distance of 110 km, a change in elevation of 1651 m, a duration of about 3 hours and 40 minutes, and an average speed of 36 km/h. During the fungal supplementation phase, we monitored the athletes before and after the second race, Gran Fondo Aprica, with a distance of 85 km, a change in elevation of 1850 m, a duration of about 4 hours, and an average speed of 33 km/h. The latter course is particularly hard due to the slopes and technical difficulties.

### 2.3. Equipment

In order to monitor energy expenditure during races, we used a Sense Wear Pro Armband TM: a commercially available device used to estimate energy expenditure [20, 21]. The Sense Wear Armband (Sense Wear Body Monitoring System by BodyMedia Pittsburgh, PA, USA) is a single device that combines five different sensors. This device is worn on the right upper arm over the triceps muscle and monitors various physiological and movement parameters: heat flux, accelerometer, galvanic skin response, skin temperature, near body temperature, and demographic characteristics including gender, age, height, and weight. These parameters are used to estimate energy expenditure utilizing proprietary equations developed by the manufacturer. Computer software (Sense Wear Professional software 6.1 by BODYMEDIA) applies activity-specific algorithms to calculate energy expenditure based on the analysis of the pattern of signals from the sensors. Metabolic equivalents (METs expressed in Kcalorie/Kg/hour) represent the energy cost of physical activities as multiples of the metabolic rate at rest [20]. Data were collected and MET were measured during the Gran Fondo races, during both the placebo and fungal supplementation phases.

### 2.4. Chemicals

All chemicals and solvents used in this study were supplied by Sigma-Aldrich Co. and kits for the ELISA test were supplied by the company DiaMetra©, 20090 Segrate-Milano, Italy.

### 2.5. Supplementation Protocol

Subjects were provided with either two commercially available capsules, one of which contained a formulation of* Ophiocordyceps sinensis* and the other a formulation of* Ganoderma lucidum*, or with placebo capsules that were identical in shape and colour. The placebo capsules contained hydroxypropyl methylcellulose (HPMC) and 18% mannitol. Every day, during the placebo phase, athletes took 5 capsules: 1 with breakfast, 2 with lunch, and 2 with dinner. Every day, during the fungal supplementation phase, athletes took 3 capsules: 1 capsule of* O*.* sinensis* with breakfast, 1 capsule of* O*.* sinensis* and 1 capsule of* G*.* lucidum* with lunch, and 1 capsule of* O*.* sinensis* and 1 capsule of* G*.* lucidum* with dinner.

The first phase of the trial protocol consisted of a month of placebo capsule intake as specified above. At the end of this phase, the athletes participated in the first bicycle race.

Immediately following the placebo phase, there was a three-month fungal supplementation phase with fungal capsule intake as specified above, up to three days before the second race, which took place at the end of this phase. The dose was increased for the final three days of this three-month period before the second race (6 capsules of* O*.* sinensis* + 2 capsules of* G*.* lucidum*/day).

*Ophiocordyceps sinensis (Cordyceps sinensis).* Ingredients per capsule are 445 mg standardized extract obtained from mycelium. Nutritional information is as follows: energy per capsule 0.12 Kcal, 33.2% polysaccharides including 7.8% *β*-glucan, 25.3% protein, 18.3% mannitol, 3.6% fat, and 0.45% adenosine.

*Ganoderma lucidum*. Ingredients per capsule are 390 mg pure extract and 3.9 mg of silicon dioxide. Nutritional information is as follows: energy per capsule 0.12 Kcal, 33% polysaccharides of which 24.6% *β*-glucans, 23% protein, 2.1% fat, and 1.5% triterpenes.

Daily fungal supplementation consisted of 1335 mg of standardized* Ophiocordyceps sinensis* extract from mycelium containing 34.71 mg of *β*-glucans and 1170 mg of* Ganoderma lucidum* pure extract, containing 95.9 mg of *β*-glucans and 4.5 mg of triterpenes.

### 2.6. Collection and Processing of Saliva and Human Serum Samples

Saliva and blood were collected just before and just after physical exertion, for each race.

The subjects were instructed to deposit saliva into a collector (Saliva Collector Devices ce DKO063-DiaMetra© Italy). The saliva was frozen at −20°C and maintained at this temperature until it arrived in the laboratory. The samples were centrifuged for 15 minutes at 655 g to remove any remaining food or possible contaminants, after which the recovered supernatant was analyzed.

Health care workers obtained venous blood samples from the volunteers using serum calibrated vacuum tubes, containing gel polymers. The collected blood was immediately centrifuged at 218 g for 15 minutes to separate the serum from the cellular component. The serum obtained was stored at −80°C.

### 2.7. Determination of Testosterone and Cortisol in Saliva by Direct Enzyme Immunoassay

The calculation of the amount of cortisol [22] and testosterone [23] in saliva were performed by means of competitive ELISA immunoenzymatic colorimetric methods (DiaMetra©, Italy).

### 2.8. Evaluation of Human Serum Antioxidant Capacity

We assessed the antioxidant effect of the athletes' serum on the stable radical cation chromophore, DPPH (1,1′-diphenyl-2-picrylhydrazyl), one of the most stable and easy-to-use synthetic free radicals. We therefore assessed the ability of the molecules present in the serum to act as scavengers of the free radical reaction, which consists in donating a hydrogen molecule to DPPH. The antioxidant capacity was calculated using a spectrophotometric measurement (spectrophotometer Shimadzu UV-1800), by the decrease in absorbance at a wavelength (*λ*) of 517 nm, which was observed following the capture of the free radical. Antioxidant activity is expressed in FRSA (free radical scavenging activity) as DPPH reduced *μ* mol/mL sample [24]. Human serum samples were treated before being analyzed: the serum was boiled at 95°C for 10 minutes to deproteinize the sample (mineralization). Subsequently, the sample was centrifuged at 2000 g for 20 minutes and the supernatant used for analysis [24].

### 2.9. Data Analysis

Descriptive statistics were expressed as means and standard error of the mean (SEM). Statistical analysis for the difference between before and after race and intergroup differences was performed using a univariate Student's paired *t* test. The level of statistical significance was set at a *P* value of <0.05 (*P value <0.05; **P value <0.01). NS means not statistically significant. Student's *t* test was applied after evaluating data assumption of normality with Shapiro-Wilk test.

## 3. Results

Seven healthy male volunteers, aged between 30 and 40 years, who are all amateur cyclists that participate in “Gran Fondo” cycling races were included in the trial.

During both placebo and fungal supplementation we measured energy expenditure during exercise by means of a Sense Wear Pro Armband TM (see Section 2): MET values for all 7 athletes ranged between 4.5 and 5 METs. All subjects in both conditions (placebo and fungal supplements) have the same energy expenditure during exercise, suggesting similar exercise intensity during races.

### 3.1. Performance Assessment

**Figure 1:**
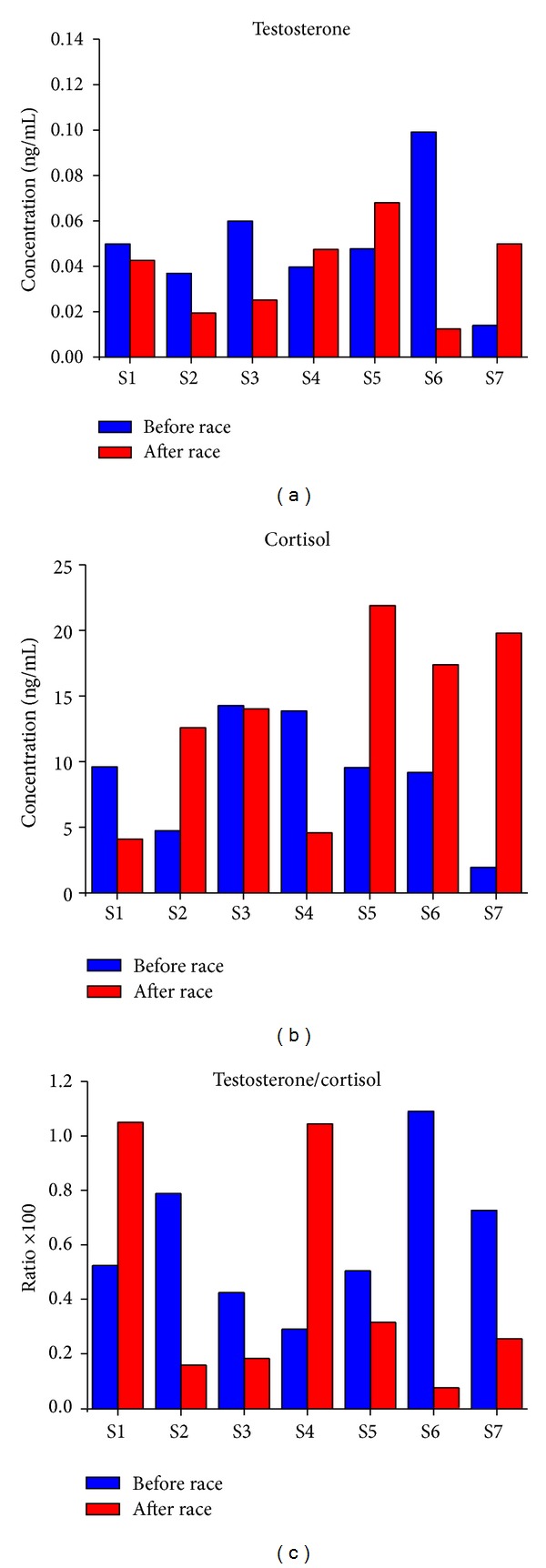
Placebo supplementation. Training condition assessment. Salivary testosterone (a), cortisol (b), and testosterone/cortisol ratio (c) in the placebo condition in 7 athletes (subjects S1–S7) just before the race (before race, blue) and just after the race (after race, red). The testosterone/cortisol ratio value is multiplied by 100.

Figure 1(a) reports salivary testosterone levels just before the race (before race) and just after the race (after race) in the placebo condition. In 4 out of the 7 athletes tested, the testosterone level decreased after the race (samples 1, 2, 3, and 6; mean value after race, 0.06 ± 0.01 ng/mL versus 0.025 ± 0.006 ng/mL value before race, mean reduction 66.7%, *n* = 4, *P* < 0.049), whereas in the other 3 athletes, the testosterone level increased after the race (samples 4, 5, and 7, mean value after race, 0.055 ± 0.006 ng/mL versus 0.034 ± 0.01 ng/mL, *n* = 3, mean increase 61.7%, *n* = 3, *t* test not significant).

Figure 1(b) shows salivary cortisol level just before and just after the race in the placebo condition. In 4 out of the 7 athletes tested, the cortisol level increased after the race (samples 2, 5, 6,and 7; mean value after race, 17.9 ± 2 ng/mL versus 6.34 ± 1.83 ng/ml value before race, mean increase, 182.3%, *n* = 4, *P* < 0.01), whereas in the other 3 athletes, the cortisol level decreased after the race (samples 1, 3, and 4; mean value after race, 7.55 ± 3.22 ng/mL versus 12.57 ± 1.49 ng/mL value before race, *n* = 3, mean decrease 39.9%, *n* = 3, *t* test not significant).

Figure 1(c) reports the testosterone/cortisol ratio before and after the race in the placebo condition (values multiplied by 100). In 5 out of the 7 athletes tested (subjects 2, 3, 5, 6, and 7), the testosterone/cortisol ratio decreased after the race compared to before the race by more than 30% (values multiplied by 100: 0.19 ± 0.04 mean value after race versus 0.7 ± 0.11 mean value before race, mean decrease 72.9%, *n* = 5, *P* < 0.0006). These 5 athletes are therefore at risk of overtraining. However, in the other 2 athletes (subjects 1 and 4) the testosterone/cortisol ratio increased after the race compared to before the race (values multiplied by 100: before race value 0.4 ± 0.11 versus 1.04 ± 0.003 after race value, increase ratio 160%, *n* = 2). More specifically, subject 1, who is a well-trained athlete, showed a slight decrease in testosterone level and a great decrease in cortisol level after race, whereas subject 4 showed a slight increase in testosterone level and a great decrease in cortisol level after race.

Therefore, we divided the athletes into two groups: well-trained (WT, subjects 1 and 4) and at risk of overtraining (OT, subjects 2, 3, 5, 6, and 7).

### 3.2. Effects of Fungal Supplements on Well-Trained Athletes

**Figure 2:**
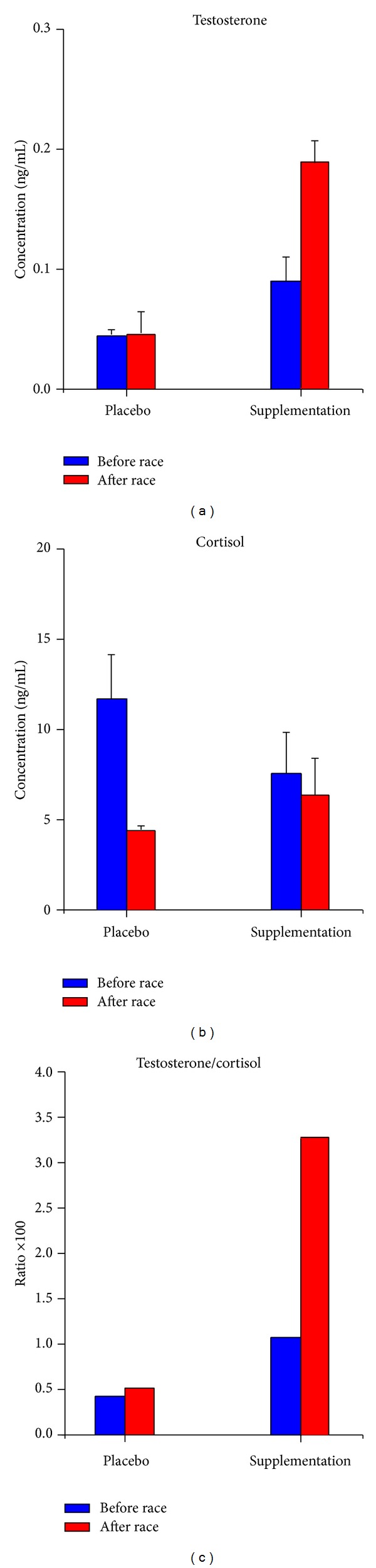
Well trained athletes.* Ophiocordyceps ganoderma* supplementation. Effects of fungal supplementation on well-trained athletes. Salivary testosterone (a), cortisol (b), and testosterone/cortisol ratio (c) after 3 months of* Ophiocordyceps ganoderma* fungal supplementation before the race (before race, blue) and after the race (after race, red). Data are reported as mean ± sd. The testosterone/cortisol ratio value is multiplied by 100.

We studied the effects of two fungal supplements on the two well-trained athletes (Figure 2). The before race basal level of salivary testosterone increased after the fungal supplementation phase that lasted 3 months (0.09 ± 0.02 ng/mL after fungal supplementation versus 0.044 ± 0.005 ng/mL in the placebo condition, Figure 2(a)). The after race testosterone level after fungal supplementation increased even more compared to the after race level in the placebo condition (0.19 ± 0.016 ng/mL after fungal supplementation versus 0.045 ± 0.002 ng/mL in the placebo condition; see Figure 2(a)).

The before race basal level of salivary cortisol decreased after the fungal supplementation phase that lasted 3 months (7.5 ± 2.3 ng/mL after fungal supplementation versus 11.7 ± 2.12 ng/mL in the placebo condition, Figure 2(b)). The after race cortisol level after fungal supplementation increased compared to the after race level in the placebo condition (6.5 ± 1.9 ng/mL after fungal supplementation versus 4.33 ± 0.22 ng/mL in the placebo condition (Figure 2(b))).

The increase in the testosterone/cortisol ratio is shown in Figure 2(c). After 3 months of fungal supplementation, the testosterone/cortisol ratio of the two athletes increased both before race (1.2 versus 0.38) and after race (ratio 2.9 versus 1.04) compared to the ratio in the placebo condition.

### 3.3. Effects of Fungal Supplements on Athletes at Risk of Overreaching/Overtraining

**Figure 3:**
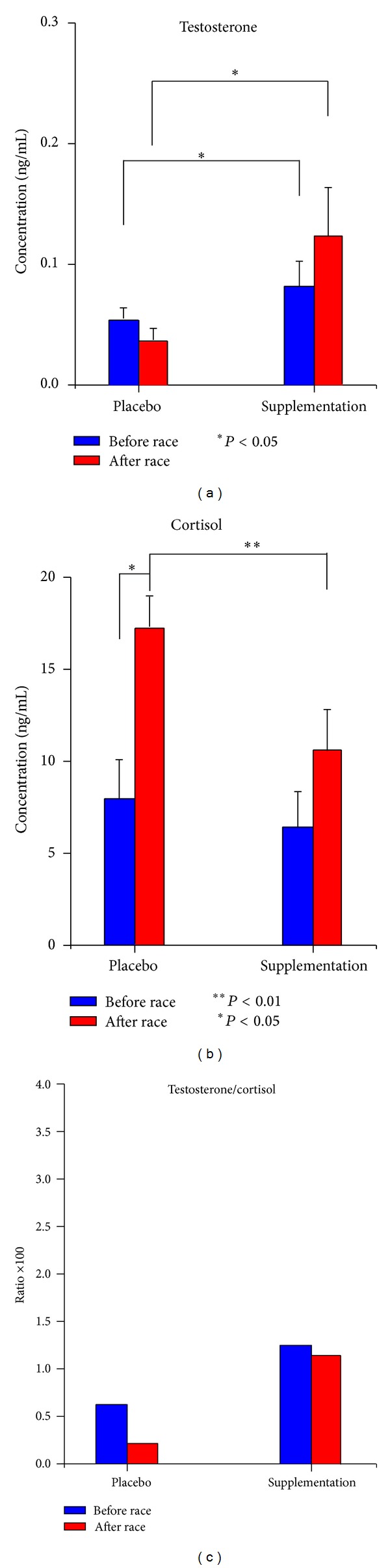
Inadequately trained athletes.* Ophiocordyceps ganoderma* supplementation. The effects of fungal supplementation on athletes at risk of overreaching/overtraining. Salivary testosterone (a), cortisol (b), and testosterone/cortisol ratio (c) after 3 months of* Ophiocordyceps ganoderma* fungal supplementation before the race (before race, blue) and after the race (after race, red). Data are reported as mean ± SEM. The testosterone/cortisol ratio value is multiplied by 100. The level of statistical significance was set at a *P* value of <0.05 (*P value <0.05; **P value <0.01). NS: not statistically significant.

Figure 3 shows the before race basal level of salivary testosterone in the placebo phase and after 3 months of fungal supplementation. The mean before race basal testosterone level increased after fungal supplementation compared to the level in the placebo condition (0.08 ± 0.02 ng/mL versus 0.052 ± 0.01 ng/mL, *n* = 5, *P* < 0.027). The after race testosterone level after fungal supplementation increased 3.4-fold compared to the after race level in the placebo condition (0.12 ± 0.04 ng/mL versus 0.035 ± 0.01 ng/mL, *P* < 0.016, Figure 3(a)).

The before race basal level of salivary cortisol after fungal supplementation is not statistically different compared to the level in the placebo condition (6.43 ± 1.75 ng/mL versus 7.92 ± 2.12 ng/mL). The after race cortisol level after fungal supplementation increased compared to the level in the placebo phase (10.58 ± 2.2 ng/mL versus 17.13 ± 1.74 ng/mL). The 2.2-fold increase of cortisol level in the placebo condition is statistically different (before race versus after race, *P* < 0.03), whereas the 1.6-fold increase after fungal supplementation is not statistically different.

We evaluated the testosterone/cortisol ratio in athletes at risk of overtraining, just before and just after the races in both the placebo phase and after 3 months of fungal supplementation (Figure 3(c)). After 3 months of fungal supplementation, the ratio values of the two athletes increased both before race (1.24 versus 0.66, values multiplied by 100) and after race (ratio 1.13 versus 0.2, values multiplied by 100).

In the placebo condition, the testosterone/cortisol ratio decreased by an average of −69.3%, suggesting that the athlete was at risk of overtraining [20], while after fungal supplementation it decreased by an average of −8.7%, so the athlete was no longer at risk of overtraining.

Four out of the 5 athletes who were shown to be at risk of overtraining in the placebo condition overcame these symptoms after fungal supplementation, whereas the testosterone/cortisol ratio in the remaining athlete in this group did not improve. After fungal supplementation, all athletes displayed an increase in the T/C ratio after race compared to before race.

### 3.4. Assessment of the Antioxidant Capacity of the Human Serum

**Figure 4:**
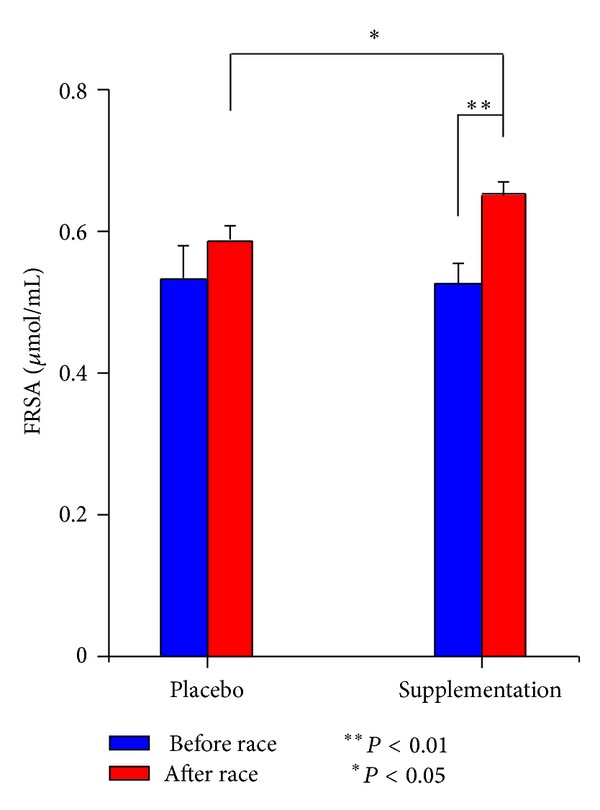
DPPH.* Ophiocordyceps ganoderma* supplementation. Human serum antioxidant capacity. Analysis of DPPH before and after the race. Measurements expressed in free radical scavenging activity (FRSA) were carried out in triplicate for each subject and are presented as mean ± mean standard error. Experimental conditions: placebo values just before the race (before race placebo) and just after the race (after race placebo); 3 months of fungal supplementation just before the race (before race* Ophiocordyceps ganoderma*) and just after the race (after race* Ophiocordyceps ganoderma*). The level of statistical significance was set at a *P* value of <0.05 (*P value <0.05; **P value <0.01). NS: not statistically significant.

The antioxidant capacity of the serum was assessed by its ability to scavenger DPPH (Figure 4) before and after race in the placebo phase and after 3 months of fungal supplementation. The antioxidant activity is expressed in FRSA (free radical scavenging activity) as DPPH reduced *μ*mol/mL sample (see Section 2).

In the placebo condition, before and after race DPPH values were not statistically significant. After 3 months of fungal supplementation, before and after race average DPPH values were statistically different (*n* = 7, *P* < 0.009, paired *t* test). Furthermore, after 3 months of fungal supplementation, FRSA after race values increased in a statistically significant manner compared to after race values in the placebo condition (*n* = 7, *P* < 0.027, paired *t* test).

## 4. Discussion

This paper investigates the problem of underperformance in athletes by referring to two different hypotheses in the pathogenesis of nonfunctional overreaching and overtraining: the hypothalamic hypothesis and the oxidative stress hypothesis.

We tested the effect of the dietary fungal supplements* Ophiocordyceps sinensis* and* Ganoderma lucidum* both on the levels of testosterone and cortisol in the saliva of road cyclists and on the scavenger ability of free radicals in their serum.

The link between hormonal status and oxidative stress has not been considered in previous publications. Excessive oxidative stress is an important mediator of a decline in steroid hormone production mediated by activated p38 mitogen-activator protein kinase (p38MAPK) [25].

The “stress response” paradigm is comparable to adrenal hyperfunction, according to which psychophysical stress and sensible activation of the hypothalamic-hypophyseal-adrenal axis cause oxidative damage. In animal models of restraint stress, adrenal hyperfunction leads to a decrease in antioxidant enzymes (SOD, Cat, GSH transferase, and reductase) and a reduction of GSH and urate in serum. In the same animal model, lipid peroxidation and carbonyl contents significantly increase in the brain, liver, and heart suggesting a causal role of stress hormones in oxidative processes induced during the adaptive response [26, 27]. Oxidative stress and inflammation are associated with fatigue and bad recovery and are risk factors for overreaching in intense exercise. Antioxidant treatment may have a positive effect on markers of oxidative stress, inflammation, and cortisol response [28]. Furthermore, there is a correlation between sex hormones and plasmatic total antioxidant capacity (TAC), and TAC significantly correlated with total testosterone [29].

The generation of reactive oxygen and nitrogen species (RONS) in response to intense exercise can occur via several pathways, including mitochondrial respiration (electron leakage from electron transport chain and subsequent production of the superoxide radical), which is the main metabolic mechanism involved in the performance of cyclists. An alteration in the redox state in favor of the generation of RONS is necessary in order to initiate signaling pathways; however, when acute or chronic free radicals are produced, the antioxidant defense system can be overwhelmed, thereby disrupting normal redox-sensitive signaling and causing a permanent shift in “redox homeostasis” [30, 31]. The development of procedures to ameliorate the production of undesirable RONS may become one of the core research areas in this field.

It is common practice for athletes to use antioxidant dietary supplements as they are known to prevent the adverse effects of exercise-induced oxidative stress, hasten the recovery of muscle function, and improve performance [32, 33]. The most commonly used supplements include vitamin E, vitamin C, *β*-carotene, coenzyme Q10, lipoic acid, N-acetylcysteine, allopurinol, quercetin, resveratrol, and several other polyphenolic compounds or a combination of the above. However, free radicals not only cause damage but they also have a role in cell signaling. RONS produced during exercise act as signals that regulate molecular events that are important in muscle cell adaptations to exercise [34]. The practical consequence is that antioxidant administration prevents such adaptations. Antioxidants, especially in high doses, have recently been shown to increase markers of exercise-induced oxidative stress or to inhibit the beneficial physiological or muscle adaptations induced by ROS [34, 35]. The practical implication is that by decreasing the effects of RONS using antioxidants, beneficial cell adaptations may be hindered during exercise.

Our data suggest that athletes may have a viable alternative to taking antioxidant dietary supplements, namely, micotherapy supplements containing nutriceuticals that do not greatly interfere with antioxidant exercise-induced adaptations, thereby improving systemic redox.

The fungal species* Ophiocordyceps sinensis* is mainly used in TCM as a tonic. It has been suggested that its antioxidant effects improve performance. The molecular mechanism responsible for the effects this fungus has on physical fitness has not yet been clarified. A pilot study [36] carried out using a fermented product of standardized* O. sinensis* on 20 healthy elderly subjects describes a 10% increase of the metabolic threshold (from 0.84 to 0.93 L/min) and of the ventilatory threshold (8.5%). In rats, oral administration of* O. sinensis* causes a statistically significant increase in the period of endurance (from 1.79 to 2.9 times) compared to the control group [36]. In the gastrocnemius muscle of the rat, higher levels of some molecules were measured, with metabolic functions at muscular level [37].

*Ophiocordyceps sinensis* is a powerful hydrogen donor, and the protection effect of oxidative damage is due to its free radical scavenger ability. The protective effects of* O. sinensis* against oxidative damage to lipids, proteins, and low-density lipoproteins (LDL) are not due to the presence of cordycepin and adenosine but due to the presence of polyphenols and flavonoids [38].

Antioxidant properties have also been reported in assays for lipid oxidation of the polysaccharide fraction from the mycelium of* O. sinensis* [39]. Sporophore water and ethanol extracts of* Ophiocordyceps sinensis* possess potent antioxidant properties [40].

The other fungal species in this study,* Ganoderma lucidum*, contains many bioactive compounds amongst which triterpenes have displayed antioxidant effects and exert a scavenging action on free radicals (superoxide, peroxyl, DPPH)* in vitro* [41, 42]. It is noteworthy that some differences between European or Chinese strains and sporophores have been registered [43, 44].

The administration of extracts of triterpenes for 1 month has been shown to enhance the activity of antioxidant enzymes such as superoxide dismutase (SOD), catalase (CAT), and glutathione peroxidase, GPX in mice,* in vivo* [41, 42]. Even at low concentrations, triterpenes have been found to be effective in preventing DNA damage and in reducing apoptosis. They also reduce the formation of reactive oxygen species (ROS) and increase the activity of endogenous antioxidant enzymes in splenic lymphocytes subjected to irradiation. This suggests that triterpenes isolated from* G. lucidum* are able to protect cells from radiation-induced damage, indicating possible applications in the field of therapy [38, 39]. Furthermore, crude ethanol and water extracts from* Ganoderma* are a rich source of antioxidant compoundssuch as phenols, ascorbic acid, *β*-carotene, and lycopene and display antioxidant properties in some different assays, namely, the 2,2-diphenyl-1-picrylhydrazyl (DPPH) radical scavenging activity and the metal chelating activity against ferrous ions.

We divided the athletes who participated in this study into two groups, according to their testosterone/cortisol ratio before and after race: well-trained athletes (2 subjects) and athletes at risk of overtraining (5 subjects).

There was no difference between the before and after race testosterone levels in the two well-trained athletes during the placebo phase, whereas there was a 2-fold increase in the before race basal testosterone level after fungal supplementation compared to the placebo condition and a 4-fold increase in the after race level. Cortisol levels did not change in a statistically significant manner before and after fungal supplementation or before and after race. The T/C ratio and scavenger activity of free radicals improved in both athletes.

After 3 months of fungal supplementation, the T/C ratio and the scavenger activity of free radicals increased in a statistically significant way in the other 5 athletes, who were potentially at risk of OTS during the placebo phase (as a result of incorrect or insufficient training), thus protecting them from overreaching and/or overtraining. In the placebo condition, after race levels of testosterone decreased compared to before race, while after race levels of cortisol increased compared to before race. After fungal supplementation, before race basal testosterone levels increased compared to before race levels in the placebo condition, and before race levels increased even more compared to the placebo condition, reaching a 3-fold increase. Before race basal cortisol levels did not change after fungal supplementation, but after race levels increased after fungal supplementation compared to levels in the placebo condition, demonstrating that the fungal supplements protected the athletes at risk of overtraining. The statistically significant increase in FRSA after race after fungal supplementation and the statistically significant difference in after race values after fungal supplementation compared to the placebo condition suggest that the 7 athletes are protected from an increase in free radical production during physical exercise.

We cannot distinguish between the two states of underperformance, because the difference is based on time before recovery and not on the degree or type of symptoms [2].

We can therefore conclude that a 3-month period of* O. sinensis* and* G. lucidum* dietary supplementation may protect endurance athletes from nonfunctional overreaching/overtraining. An interesting future development of this research would be to analyze inflammatory parameters in order to understand the role fungal supplementation plays on the immune system. The study should continue to select standardized fungal dietary supplements, but it should be expanded to include a larger number of endurance athletes, due to the variability in their athletic condition [45].

## Figures and Tables

**Table 1:** Age, weight, height, body mass index (BMI), and related SD of amateur cyclists who participated in the trial.

	Age (years)	Weight (Kg)	Height (cm)	BMI (Kg/m^2^)
Mean	39.7	73.8	176.7	23.7
SD	5.0	6.1	5.5	1.6
*n*	7	7	7	7
